# NUDT21 Promotes Tumor Growth and Metastasis Through Modulating SGPP2 in Human Gastric Cancer

**DOI:** 10.3389/fonc.2021.670353

**Published:** 2021-09-30

**Authors:** Yong Zhu, Rumeng Zhang, Ying Zhang, Xiao Cheng, Lin Li, Zhengsheng Wu, Keshuo Ding

**Affiliations:** ^1^Department of Pathophysiology, School of Basic Medicine, Anhui Medical University, Hefei, China; ^2^Department of Pathology, School of Basic Medicine, Anhui Medical University, Hefei, China; ^3^Department of Oncology of the First Affiliated Hospital, Division of Life Science and Medicine, The CAS Key Laboratory of Innate Immunity and Chronic Disease, University of Science and Technology of China, Hefei, China; ^4^Department of Pathology, The First Affiliated Hospital of Anhui Medical University, Hefei, China

**Keywords:** NUDT21, SGPP2, gastric cancer, proliferation, metastasis

## Abstract

Gastric cancer is one of the major malignancies with poor survival outcome. In this study, we reported that NUDT21 promoted cell proliferation, colony formation, cell migration and invasion in gastric cancer cells. The expression levels of NUDT21 were found to be much higher in human gastric cancer tissues compared with normal gastric tissues. NUDT21 expression was positively correlated with tumor size, lymph node metastasis and clinical stage in gastric cancer patients. High level of NUDT21 was associated with poor overall survival (OS) rates in gastric cancer patients. The expression levels of NUDT21 were also much higher in gastric cancer tissues from patients with distant metastasis compared with those of patients without distant metastasis. Moreover, forced expression of NUDT21 in gastric cancer cells promoted tumor growth and cell proliferation in xenograft nude mice, and depletion of NUDT21 in gastric cancer cells restrained lung metastasis *in vivo*. Through high throughput RNA-sequencing, SGPP2 was identified to be positively regulated by NUDT21 and mediated the tumor promoting role of NUDT21 in gastric cancer cells. Therefore, NUDT21 played an oncogenic role in human gastric cancer cells. NUDT21 could be considered as a novel potential target for gastric cancer therapy.

## Introduction

Gastric cancer is one of the major malignancies with high morbidity worldwide (1). Effective specific diagnosis, prognostic markers and therapeutic targets for gastric cancer remain limited. The median 5-year survival rates of patients with advanced gastric cancer are less than 20% (2). Currently, the optimal remedy for gastric cancer is surgical resection, supplemented by adjuvant chemotherapy or radiotherapy (3). However, the recurrence rate of gastric cancer is still high and the prognosis of gastric cancer patients is poor (4, 5). It deserves further study about the intrinsic molecular mechanisms involved in tumorigenesis, proliferation and metastasis of gastric cancer to improve clinical outcomes.

NUDT21 (nudix hydrolase 21), a complex of RNA-binding proteins, has been reported to be involved in alternative polyadenylation (APA) (6, 7). Justin et al. reported that NUDT21 was a new post-transcriptional regulator of cell fate, a direct link was established between alternative polyadenylation and chromatin signaling. Moreover, NUDT21 plays an important physiological function in the occurrence and development of many diseases (8). For example, the study of Xiao-Lang et al. proved that abnormal expression of NUDT21 promoted preeclampsia by targeting the 3’-UTR of EZH2 mRNA (9). There are reports that NUDT21-mediated Glutaminase isoform switching promotes hematopoietic stem cell metabolism upon stress (10). In addition, NUDT21-mediated high-mobility AT-hook 2 (HMGA2) 3’-UTR replacement polyadenylic acid damages the stemness of human tendon stem cells (11). As reported previously, NUDT21 played oncogenic roles in human pancreatic ductal adenocarcinoma cells and leukemia cells (12–14). However, in our previous study, we have found NUDT21 inhibits cell proliferation and metastasis in human hepatocellular carcinoma and breast cancer (15, 16). Nevertheless, the role of NUDT21 in human gastric cancer cells remains unclear.

In this study, we demonstrated that NUDT21 was high expressed in human gastric cancer tissues/cells compared with normal gastric tissues/cells. The expression levels of NUDT21 were also higher in gastric cancer tissues from patients with distant metastasis compared with the tissues from patients without distant metastasis. Gastric cancer patients with high expression of NUDT21 showed poor clinicopathological parameters and overall survival (OS) rates. Depletion of NUDT21 decreased both cell proliferation and metastasis in gastric cancer cells. Forced expression of NUDT21 enhanced cell oncogenic futures of gastric cancer cells. NUDT21 but also promoted tumor growth and metastasis in nude mice. Through high throughput RNA-sequencing, an important oncogene SGPP2 (sphingosine-1-phosphate phosphatase 2, also known as SPP2) was found to be positively regulated by NUDT21 and mediated the tumor promoting role of NUDT21 in gastric cancer cells. Therefore, we provided evidence that NUDT21 played an important oncogenic role in human gastric cancer. NUDT21 could possibly be perceived as a novel target for gastric cancer therapy.

## Materiials And Methods

### Clinical Samples

In this study, 70 cases of paraffin-embedded gastric cancer tissues and 70 normal gastric tissues were collected at the Department of Pathology in the First Affiliated Hospital of Anhui Medical University (Hefei, Anhui, China). These tissues were from patients who underwent surgical resection between 2012 and 2015, and gastric cancer tissues were not paired with normal gastric tissues. The clinicopathological parameters of these gastric cancer patients were collected for correlation analysis. Moreover, these 70 gastric cancer patients were followed up for more than 5 years, and their OS rates were collected. We performed this work according to The Code of Ethics of the World Medical Association (Declaration of Helsinki). This work has been approved by the Institutional Review Board of Anhui Medical University and the informed consent of all patients has been obtained.

### Immunohistochemistry

Immunohistochemistry (IHC) was performed to examine the expression of NUDT21 protein in paraffin sections of gastric cancer/normal gastric tissues and the expression of Ki-67 protein in paraffin sections of tumors in mice, essentially as described in former study by using an Ultra Sensitive-SP kit (Maixin-Bio, Fuzhou, China) (17, 18). Rabbit polyclonal antibody against NUDT21 (both 1:100, Proteintech Group, Inc., Chicago, USA) and mouse monoclonal antibody against Ki-67 (1:1, Zhongshan Goldenbrige Biotechnology Co, Beijing, China) were used. The stained sections were evaluated using an Olympus microscope (Olympus America, Inc., Melville, NY). For NUDT21 analysis, tissue samples with 10% or more positive stained cells were designated as NUDT21 positive; tissue samples with less than 10% positive stained cells were designated as NUDT21 negative.

### Cells Lines and Cell Culture

Human gastric cancer cell lines (AGS, BGC-823, SGC-7901, HGC-27, MKN-28) were from the American Type Culture Collection (ATCC, Manassas, VA). The normal gastric cell line GES-1 was from the Chinese Academy of Sciences (Shanghai, China). All of these cells were cultured in a 37°C incubator containing 5% CO_2_ as recommended.

### Western Blot

Cells were lysed with lysis buffer, centrifuged and extracted, and the proteins were separated by 10% SDS-PAGE, and then transferred to a PVDF membrane (Merck Millipore, Darmstadt, Germany). It was blocked in 5% skim milk at room temperature for 40 minutes, the membranes were incubated with primary antibodies at room temperature for 2 hours, and then washed with Tween-20-containing phosphate buffer for 3 times. Secondary antibody incubation was performed for 1 hour. After washing for 3 times with PBST, Pierce ECL substrate (Advansta) and ChemiDoc Mp System (BioRad) were used to detect the immunoreactive signal. ImageJ software (National Institutes of Health) was used to analyze the optical density of the bands. Rabbit polyclonal antibody against NUDT21, Bcl-2, CCNE1 (all 1:1000, Proteintech Group, Inc., Chicago, USA), rabbit polyclonal antibody against SGPP2 (1:1000, Sigma) and mouse β-actin monoclonal antibody (1:5000, Sigma) were used. The whole gel images were shown in **Supplementary Figure 3**.

### RT-qPCR

RT-qPCR was performed essentially as described in former study by using SYBR Green Master Mix (Applied Biosystems) (17, 19). Trizol (Invitrogen) was used to extract total RNA. Prime Script RT reagent kit (TaKaRa Bio, Inc., Otsu, Japan) was used to synthesize single-stranded cDNA. The relative expression levels of RNA were analyzed using the 2^–ΔΔCT^ method. GAPDH was detected as control. The primers used were: NUDT21: 5’- GGTCACTCAGTTCGGCAACAA -3’ (forward) and 5’- CTCATGCGCTGAAATCTGGC -3’ (reverse); SGPP2: 5’- TGTGTTGGGACTGGTGATGG -3’ (forward) and 5’- TGTAGGTGGTGAGTGGTGGG -3’ (reverse); c-Myc: 5’- TGAGGAGACACCGCCCAC -3’ (forward) and 5’- CAACATCGATTTCTTCCTCATCTTC -3’ (reverse); Bcl-2: 5’- AGTGGGATGCGGGAGATGT -3’ (forward) and 5’- CGGGCTGGGAGGAGAAGA -3’ (reverse); CCND1: 5’- TGAACTACCTGGACCGCTTC -3’ (forward) and 5’- CCACTTGAGCTTGTTCACCA -3’ (reverse); CCNE1: 5’- GCCAGCCTTGGGACAATAATG -3’ (forward) and 5’- CTTGCACGTTGAGTTTGGGT -3’ (reverse); p53: 5’- CAGCACATGACGGAGGTTG -3’ (forward) and 5’- TCATCCAAATACTCCACACGC -3’ (reverse); PTEN: 5’- TGGATTCGACTTAGACTTGACC -3’ (forward) and 5’- AGGATATTGTGCAACTCTGCAA -3’ (reverse); GAPDH: 5’- TGACTTCAACAGCGACACCCA -3’ (forward) and 5’- CACCCTGTTGCTGTAGCCAAA -3’ (reverse).

### Plasmid Constructs and RNA Oligonucleotides Transfection

In this study, we used the mammalian expression vector pIRESneo3 (Invitrogen) to construct NUDT21 and SGPP2 overexpression plasmids. The transcript of the NUDT21 coding sequence (GenBank accession number NM_007006.3) was cloned into pIRESneo3 and designated as NUDT21#OE. The SGPP2 coding sequence transcript (GenBank accession number NM_001320833.2) was cloned into pIRESneo3 and designated as SGPP2#OE. shRNAs including sh-NUDT21#1, sh-NUDT21#2, sh-ctrl and siRNAs including si-SGPP2 and si-NC were synthesized in GenePharma (Shanghai, China). Plasmid constructs and RNA oligonucleotides were transfected using lip2000 (QIAGEN). Specific shRNA and siRNA sequences were: NUDT21: 5’- GGTCACTCAGTTCGGCAACAA -3’ (forward) and 5’- CTCATGCGCTGAAATCTGGC-3’ (reverse); SGPP2: 5’- TGTGTTGGGACTGGTGATGG -3’ (forward) and 5’- TGTAGGTGGTGAGTGGTGGG -3’ (reverse); GAPDH: 5’- TGACTTCAACAGCGACACCCA -3’ (forward) and 5’- CACCCTGTTGCTGTAGCCAAA -3’ (reverse).

### Cell Functional Assays

MTT assay, cell colony formation assay, EdU assay, cell migration assay and cell invasion assay were performed as described in previous study (17, 18, 20, 21). Briefly, for MTT assay, 1000 cells per well were inoculated in 96-well plates, and MTT examination was performed every day for 5 days. A microplate reader (BioTek, Vermont, USA) was used to detect the absorbance at 570 nm. For cell colony formation assay, 1000 cells per well were inoculated in 6-well plates and cell colony formation was examined after 2 weeks. For EdU assay, the EdU test kit (Ribobio, Guangzhou, China) was used. For cell migration and invasion assays, cells were seeded into the upper chamber with (migration assay) or without (invasion assay) matrigel. Transferred cells were calculated after 24-48 hours. Images were taken using an Olympus IX-70 microscope.

### Xenograft Analyses

For nude mice subcutaneous injection, cells (500×10^4^ per site per 125μl) were injected subcutaneously into the dorsal side of 4-week-old BALB/c-nu/nu mice (GemPharmatech Co., Ltd., China). Both cells were injected into 6 mice (12 sites) respectively, and significant tumors formed after about a week. Length and width of the tumors were measured every 5 days. Tumor volume was calculated according to the formula: Volume (mm³) = L×W^2^×Π/6. After 30 days, these mice were sacrificed and tumors were harvested. The weight of the tumors was measured, and then these tumors were made into paraffin sections and the Ki-67 protein levels were examined by immunohistochemistry. For nude mice tail vein injection, cells (500×10⁶ cells in 250μL PBS) were injected into 4-week-old BALB/c-nu/nu mice by tail vein injection. Each group contained 8 mice. After about 40 days, these mice were sacrificed and their lungs were collected and prepared for histological examination. Five random hematoxylin and eosin stained sections of each sample were examined, and the lung micrometastasis was calculated. The animal experiments in this study were conducted in accordance with the guidelines of the Institutional Animal Care and Use Committee (available from www.iacuc.org). Local agency approval of the Animal Ethics Committee of Anhui Medical University has been obtained before work.

### RNA-Sequencing

To explore the downstream mechanisms involved in the role of NUDT21 in gastric cancer cells, BGC-823-sh-NUDT21#1 and BGC-823-sh-ctrl cells were harvested and mRNA sequencing was carried out in Kangcheng Biotechnology Company (Shanghai, China).

### Statistical Analyses

Each experiment in this study was performed for at least 3 times, and the figures and tables were the average results. Chi-square test was used to analyze the expression levels of NUDT21 in clinical tissues, the correlation between NUDT21 expression and clinicopathological parameters in gastric cancer patients, and the lung metastasis in mice. Unpaired two-tailed t-test was used in RT-qPCR, MTT assay, cell colony formation assay, EdU assay, cell migration assay, cell invasion assay, tumor growth curves and tumor weight in mice and the Ki-67 examination. Kaplan–Meier curves were used to analyze the OS rates in gastric cancer patients, and Log rank test was used. The correlation between NUDT21 and SGPP2 expression was analyzed by Pearson correlation analysis. *P*<0.05 indicated that the difference was statistically significant.

## Results

### Expression of NUDT21 in Human Gastric Cancer/Normal Gastric Tissue Samples and Cell Lines

**Figure 1:**
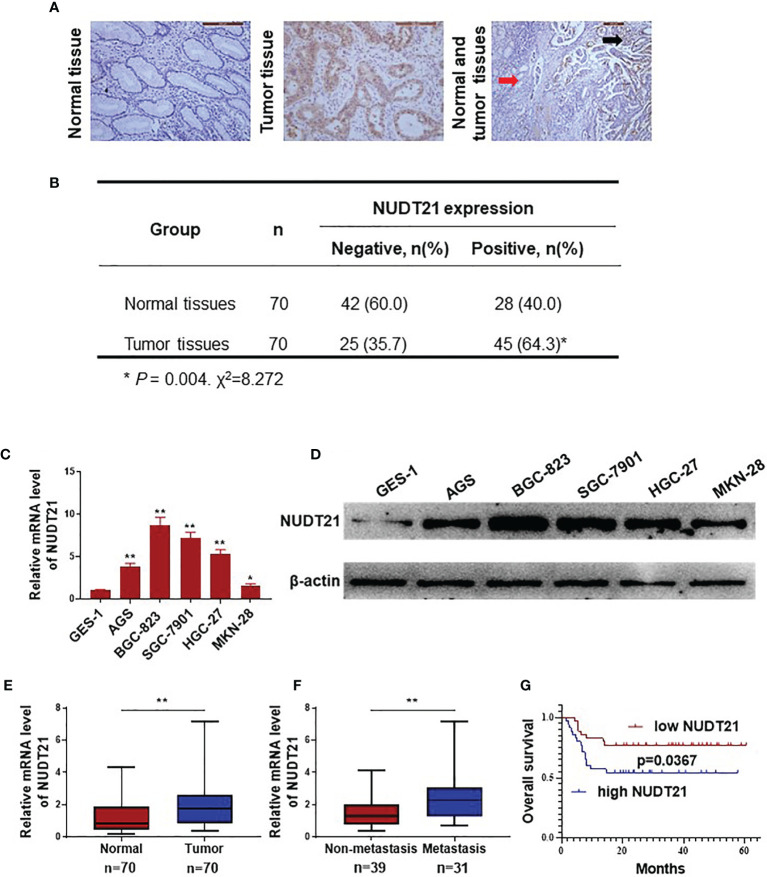
Expression of NUDT21 in human gastric cancer/normal gastric tissue samples and cell lines. **(A, B)** Protein levels of NUDT21 in 70 gastric cancer tissues and 70 normal gastric tissues were examined by immunohistochemistry. The magnifications were 200 and 100 respectively. Red arrows: normal gastric tissue; black arrows: gastric cancer tissue. **(C)** Relative mRNA levels of NUDT21 in gastric cancer cells AGS, BGC-823, SGC-7901, HGC-27, MKN-28 and normal gastric cell GES-1 were examined by RT-qPCR. GAPDH was used as control. **(D)** Protein levels of NUDT21 in gastric cancer cells AGS, BGC-823, SGC-7901, HGC-27, MKN-28 and normal gastric cell GES-1 were examined by western blot. β-Actin was used as control. **(E)** mRNA levels of NUDT21 in 70 gastric cancer tissues and 70 normal gastric tissues were examined by RT-qPCR. **(F)** mRNA levels of NUDT21 in 31 gastric cancer tissues from patients with distant metastasis and 39 gastric cancer tissues from patients without distant metastasis were analyzed by RT-qPCR. **(G)** Kaplan-Meier curves showed the overall survival (OS) rates in gastric cancer patients with high and low levels of NUDT21. *P < 0.05; **P < 0.01.

In this study, 70 gastric cancer tissues and 70 normal gastric tissues from patients were collected and immunohistochemistry (IHC) assay was performed to determine the protein level of NUDT21. As shown in **Figure 1A**, NUDT21 protein was mainly located in the cytoplasm of gastric cancer cells and glandular epithelial cells. The expression levels of NUDT21 were extremely higher in gastric cancer tissues (64.3%) compared with normal gastric tissues (40.0%) (*P*<0.01) (**Figures 1A, B**). The mRNA levels of NUDT21 in normal gastric cell line GES-1 and gastric cancer cell lines AGS, BGC-823, SGC-7901, HGC-27, MKN-28 were examined by RT-qPCR. As shown in **Figure 1C**, the mRNA levels of NUDT21 were extremely higher in gastric cancer cells compared with normal gastric cells (*P*<0.05). Moreover, the protein levels of NUDT21 in gastric cancer cells AGS, BGC-823, SGC-7901, HGC-27, MKN-28 were also much higher compared with normal gastric cell line GES-1 as determined by western blot (**Figure 1D**). For further study, the mRNA levels of NUDT21 in the 70 gastric cancer tissues and 70 normal gastric tissues were also examined. Concordantly, the mRNA levels of NUDT21 were significantly higher in the gastric cancer tissues compared with normal gastric tissues (**Figure 1E**). Moreover, the mRNA levels of NUDT21 were dramatically higher in gastric cancer tissues from patients with distant metastasis compared with gastric cancer tissues from patients without distant metastasis (**Figure 1F**). Therefore, the expression levels of NUDT21 were higher in human gastric cancer tissues/cells compared with normal gastric tissues/cells.

### High Level of NUDT21 Was Associated With Poor Clinicopathological Features and Survival Rates in Gastric Cancer Patients

In these 70 gastric cancer patients, their clinicopathological features (including age, gender, tumor size, lymph node metastasis, histology grade and clinical stage) were collected and the correlation between NUDT21 expression levels and the clinicopathological features were analyzed. As shown in **Table 1**, the expression of NUDT21 was positively correlated with tumor size (*P*=0.017), lymph node metastasis (*P*=0.020) and clinical stage (*P*=0.006). However, there was no statistically significant correlation between NUDT21 expression with patients´ age, gender or histology grade (all *P*>0.05). Moreover, these 70 gastric cancer patients were followed up for more than 5 years, and the correlation between NUDT21 expression and patient overall survival (OS) rates was analyzed through Kaplan-Meier analysis. Patients with low expression level of NUDT21 showed significantly lower OS rates compared with patients with high expression level of NUDT21 (*P*=0.0367) (**Figure 1G**). Therefore, high level of NUDT21 was associated with poor clinicopathological features and survival rates in gastric cancer patients.

**Table 1:** Association of NUDT21 expression with clinicopathological features in gastric cancer patients.

Parameter	NUDT21 expression [n (%)]			*P*	χ^2^
	*n*	Negative	Positive		
Age (years)					
≤60	32	11 (34.4)	21 (65.6)	0.830	0.046
>60	38	14 (36.8)	24 (63.2)		
Gender					
male	39	13 (33.3)	26 (66.7)	0.641	0.217
female	31	12 (38.7)	19 (61.3)		
Tumor size (cm)					
≤5	43	20 (46.5)	23 (53.5)	0.017	5.661
>5	27	5 (18.5)	22 (81.5)		
Lymph node metastasis					
No	24	13 (54.2)	11 (45.8)	0.020	5.416
Yes	46	12 (26.1)	34 (73.9)		
Grade					
I-II	43	18 (41.9)	25 (58.1)	0.176	1.834
III	27	7 (25.9)	20 (74.1)		
Stage					
I-II	22	13 (59.1)	9 (40.9)	0.006	7.636
III-IV	48	12 (25.0)	36 (75.0)		

### NUDT21 Stimulated Cell Proliferation, Migration and Invasion of Human Gastric Cancer Cells

**Figure 2:**
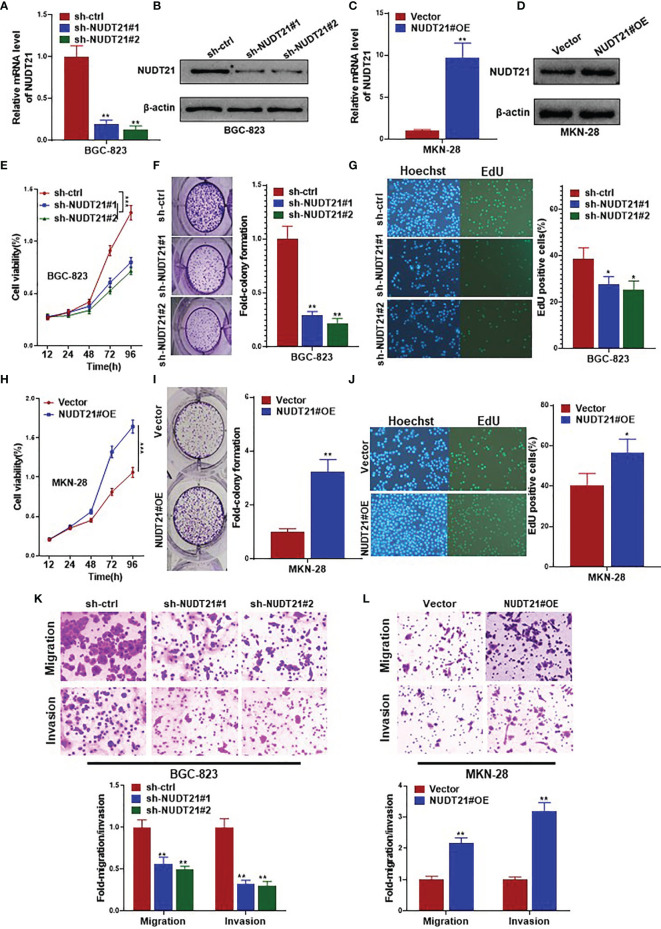
NUDT21 stimulated cell proliferation, migration and invasion of human gastric cancer cells. BGC-823 cells were transfected with sh-NUDT21#1, sh-NUDT21#2 or sh-ctrl. MKN-28 cells were transfected with the NUDT21 overexpression plasmids (NUDT21#OE) and Vector control plasmids (Vector). **(A, C)** Relative mRNA levels of NUDT21 were examined by RT-qPCR. GAPDH was used as control. **(B, D)** Protein levels of NUDT21 were detected by western blot. β-Actin was used as control. **(E, H)** MTT assay was carried out to evaluate cell viability. **(F, I)** Cell colony formation assay. Cell colony numbers were calculated after 14 days. **(G, J)** EdU assay was performed to evaluate cell proliferation. **(K, L)** Cell migration and invasion assays were carried out to examine cell metastasis. *P < 0.05; **P < 0.01; ***P < 0.001.

Gastric cancer cell lines BGC-823 and MKN-28 were selected to examine the functional role of NUDT21 in gastric cancer cells. As shown in **Figures 1C, D**, among the five gastric cancer cell lines, the basal level of NUDT21 was highest in BGC-823 cells, and was lowest in MKN-28 cells. Therefore, we selected BGC-823 cells for NUDT21 depletion study, and selected MKN-28 cells for NUDT21 overexpression study. After transfected with NUDT21 shRNAs (designated as sh-NUDT21#1 and sh-NUDT21#2), both mRNA and protein levels of NUDT21 decreased significantly in BGC-823 cells (**Figures 2A, B**). In monolayer culture, MTT assay showed a significant decrease of cell viability after transfected with sh-NUDT21#1 or sh-NUDT21#2 in BGC-823 cells within 5 days (**Figure 2E**). The shRNA mediated depletion of NUDT21 significantly reduced cell colony formation in BGC-823 cells (**Figure 2F**). Moreover, in EdU assay, sh-NUDT21#1 or sh-NUDT21#2 extremely reduced the numbers of proliferating cells compared with control shRNA (sh-ctrl) in BGC-823 cells (**Figure 2G**). In addition, as determined by cell migration and invasion assays, BGC-823 cells after transfected with sh-NUDT21#1 or sh-NUDT21#2 showed both significant decreased cell migration and invasion compared with control (**Figure 2K**).

On the other hand, the expression levels (both mRNA and protein) of NUDT21 increased significantly after stably transfected with NUDT21 overexpression plasmids (NUDT21#OE) compared with control (Vector) in MKN-28 cells (**Figures 2C, D**). As shown in **Figure 2H**, cell viability of MKN-28 cells increased significantly after transfected with NUDT21#OE within 5 days compared with control Vector. Concordantly, forced expression of NUDT21 enhanced cell colony formation in MKN-28 cells as determined by cell colony formation assay (**Figure 2I**). The number of proliferating cells in MKN-28 cells transfected with NUDT21#OE increased significantly compared with control as examined by EdU assay (**Figure 2J**). In addition, forced expression of NUDT21 significantly promoted both cell migration and invasion in MKN-28 cells (**Figure 2L**).

Therefore, NUDT21 stimulated cell proliferation, migration and invasion in human gastric cancer cells.

### NUDT21 Promoted Xenograft Growth and Metastasis of Human Gastric Cancer Cells

**Figure 3:**
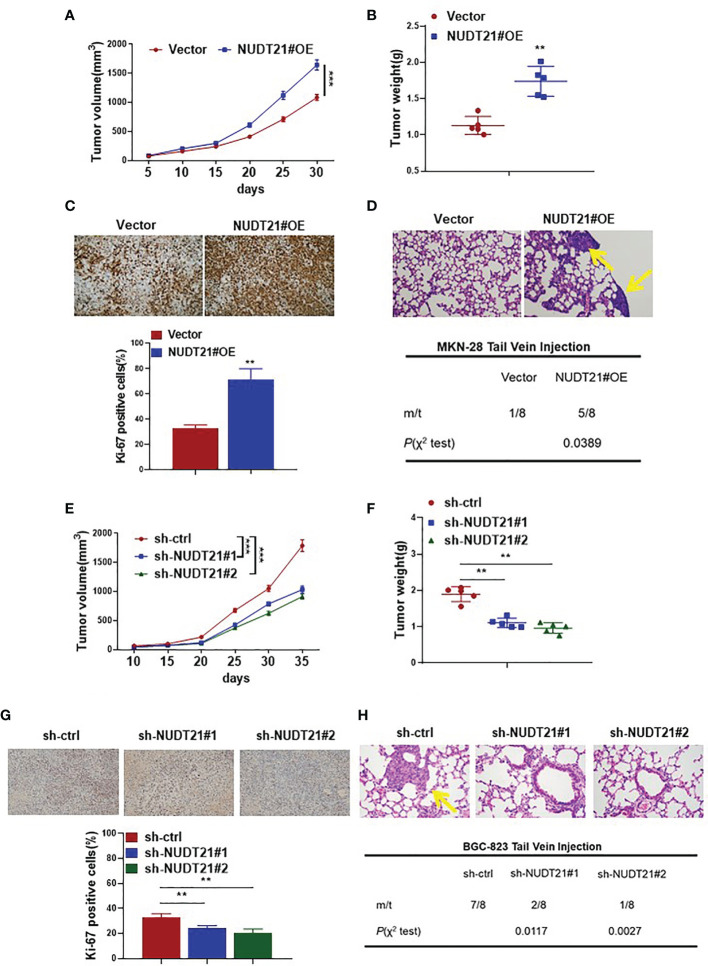
NUDT21 promoted tumor growth and metastasis of gastric cancer cells in nude mice. MKN-28-NUDT21#OE/MKN-28-Vector cells and BGC-823-sh-ctrl/BGC-823-sh-NUDT21#1/BGC-823-sh-NUDT21#2 cells were subcutaneously injected into the dorsal side of nude mice. Each group contained 6 mice. **(A, E)** Tumor volumes were evaluated every 5 days for 30 days, and tumor growth curves were analyzed. **(B, F)** Weights of the tumors were measured. **(C, G)** Ki-67 staining in xenograft tumor sections by immunohistochemistry. MKN-28-NUDT21#OE/MKN-28-Vector cells and BGC-823-sh-ctrl/BGC-823-sh-NUDT21#1/BGC-823-sh-NUDT21#2 cells were injected into the venous circulation of nude mice. Each group contained 8 mice. After about 40 days, the mice were sacrificed and their lungs were collected. **(D, H)** Hematoxylin and eosin staining of lung sections. The arrows pointed to tumor micrometastasis. Lung metastasis of tumor in these mice was calculated. **P < 0.01; ***P < 0.001.

To determine the effect of NUDT21 on tumor growth *in vivo*, MKN-28 cells stably transfected with NUDT21 overexpression plasmids (NUDT21#OE) or control vector plasmids (Vector) were injected subcutaneously into the dorsal side of nude mice. Tumor sizes were measured every 5 days. The tumor growth curves showed that the generated MKN-28-NUDT21#OE tumors grew much faster than the MKN-28-Vector tumors (**Figure 3A**). After 30 days, these mice were sacrificed and the tumors were harvested. The average weight of tumors formed by MKN-28-NUDT21#OE cells was significantly higher than that of tumors formed by MKN-28-Vector cells (*P*<0.01) (**Figure 3B**). Moreover, these tumors were made into paraffin sections and the Ki-67 protein levels were examined by immunohistochemistry. As shown in **Figure 3C**, the Ki-67-positive cell population in tumors formed by MKN-28-NUDT21#OE cells was extremely higher compared with tumors formed by MKN-28-Vector cells (*P*<0.01). Meantime, we tested the effect of NUDT21 on tumor metastasis *in vivo* by nude mice tail vein injection of MKN-28-NUDT21#OE and MKN-28-Vector cells. Forty days after cell injection, these mice were sacrificed and their lungs were collected for histological examination. Five random sections of each mouse lung were examined for tumor micrometastasis. More mice injected with MKN-28-NUDT21#OE cells (5 out of 8) were observed obvious lung micrometastasis compared with mice injected with MKN-28-Vector cells (1 out of 8) (*P*=0.0389) (**Figure 3D**).

Moreover, NUDT21 shRNAs or control shRNA transfected BGC-823 cells (designated as BGC-823-sh-NUDT21#1, sh-NUDT21#2, and sh-ctrl respectively) were also injected subcutaneously into the dorsal side and into tail vein respectively to examine the effect of NUDT21 on tumor growth and metastasis *in vivo.* Concordantly, both BGC-823-sh-NUDT21#1 and BGC-823-sh-NUDT21#2 derived tumors grew much slower than the BGC-823-sh-ctrl tumors (**Figure 3E**). The average weights of tumors formed by BGC-823-sh-NUDT21#1 or BGC-823-sh-NUDT21#2 cells were significantly lower than that of tumors formed by BGC-823-sh-ctrl cells (both *P*<0.01) (**Figure 3F**). The Ki-67-positive cell populations in tumors formed by BGC-823-sh-NUDT21#1 or BGC-823-sh-NUDT21#2 cells were extremely lower compared with tumors formed by BGC-823-sh-ctrl cells (both *P*<0.01) (**Figure 3G**). Fewer mice injected with BGC-823-sh-NUDT21#1 cells (2 out of 8) or BGC-823-sh-NUDT21#2 cells (1 out of 8) were observed obvious lung micrometastasis compared with mice injected with BGC-823-sh-ctrl cells (7 out of 8) (*P*=0.0117 and *P*=0.0027 respectively) (**Figure 3H**).

Therefore, NUDT21 promoted gastric cancer cell proliferation, tumor growth and tumor metastasis *in vivo*.

### NUDT21 Regulated the Expression of SGPP2 in Gastric Cancer Cells

**Figure 4:**
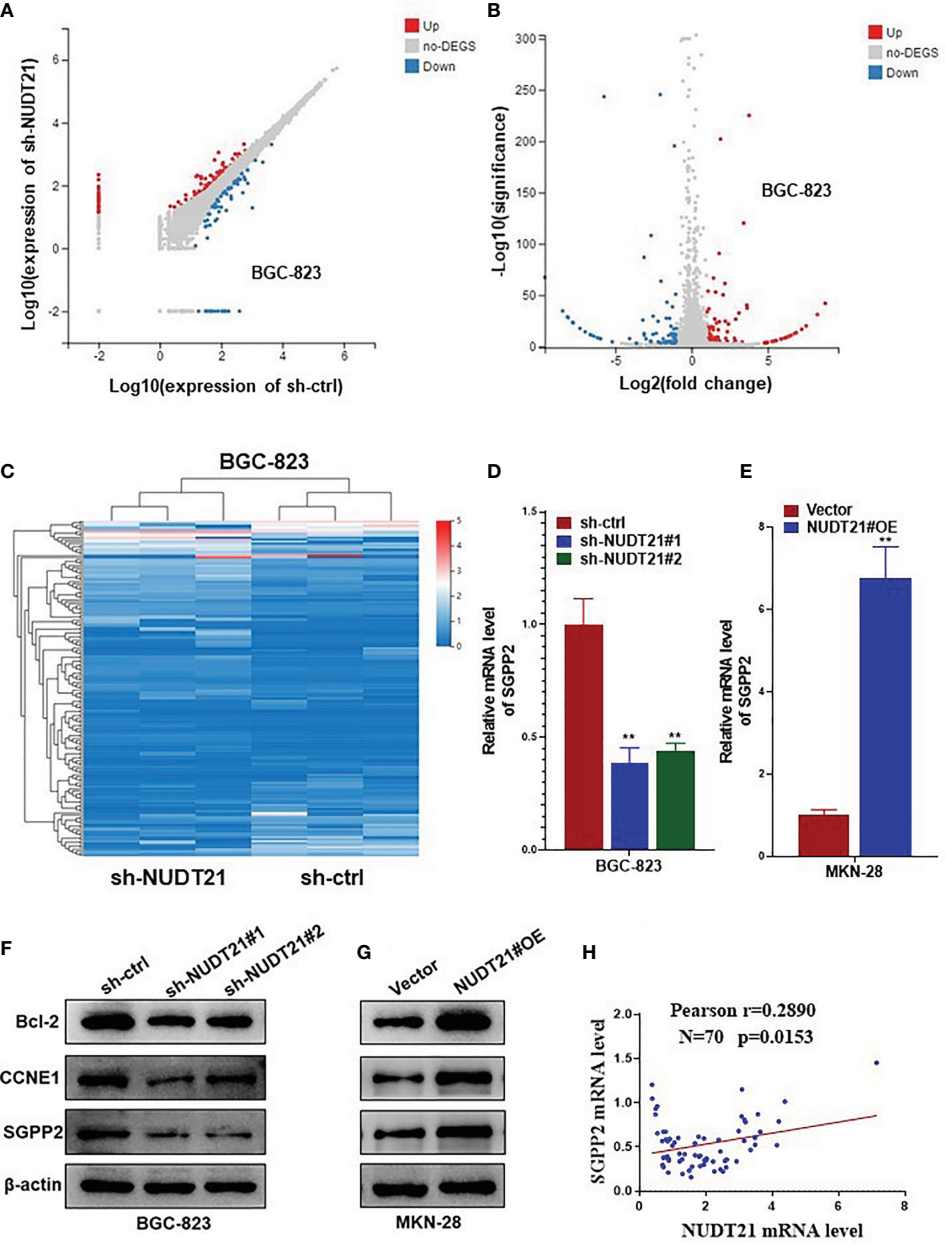
NUDT21 regulated the expression of SGPP2 in gastric cancer cells. mRNA sequencing was performed in BGC-823-sh-NUDT21#1 and BGC-823-sh-ctrl cells. **(A, B)** Scatter plots showed the mRNAs with significant change between BGC-823-sh-NUDT21#1 and BGC-823-sh-ctrl cells. **(C)** Hierarchical clustering analysis. **(D)** mRNA levels of SGPP2 in BGC-823 cells after transfected with sh-NUDT21#1, sh-NUDT21#2 or sh-ctrl were examined by RT-qPCR. **(E)** mRNA levels of SGPP2 in MKN-28 cells after transfected with NUDT21#OE or Vector were examined by RT-qPCR. **(F)** Protein levels of Bcl-2, CCNE1 and SGPP2 in BGC-823 cells after transfected with sh-NUDT21#1, sh-NUDT21#2 or sh-ctrl were examined by western blot. **(G)** Protein levels of Bcl-2, CCNE1 and SGPP2 in MKN-28 cells after transfected with NUDT21#OE or Vector were examined by western blot. For RT-qPCR, GAPDH was used as control. For western blot, β-Actin was used as control. **(H)** The mRNA levels of NUDT21 and SGPP2 in the 70 gastric cancer tissues were examined by RT-qPCR, and the correlation between NUDT21 and SGPP2 expression was analyzed by Pearson correlation analysis. **P < 0.01.

To explore the downstream mechanisms involved in the tumor promoting role of NUDT21 in gastric cancer cells, we performed high throughput RNA-sequencing to find gene differential expression between BGC-823-sh-NUDT21#1 and BGC-823-sh-ctrl cells. **Figures 4A** **–C** showed the differential gene expression profile in BGC-823-sh-NUDT21#1 and BGC-823-sh-ctrl cells. Among these genes, the mRNA level SGPP2 was found to be significantly lower in BGC-823-sh-NUDT21#1 cells compared with BGC-823-sh-ctrl cells (in fact, several candidate genes were examined to be regulated by NUDT21, and SGPP2 was examined to mediate the tumor promoting role of NUDT21 in gastric cancer cells). To confirm this result, the mRNA and protein levels of NUDT21 in BGC-823 cells after transfected with sh-NUDT21#1, sh-NUDT21#2 and sh-ctrl were examined by RT-qPCR and western blot respectively. Concordant with the RNA-sequencing result, both mRNA and protein levels of SGPP2 decreased dramatically in BGC-823 cells after transfected with sh-NUDT21#1 or sh-NUDT21#2 compared with cells transfected with sh-ctrl (**Figures 4D, F**). Moreover, the mRNA and protein levels of SGPP2 in MKN-28 cells after transfected with NUDT21#OE and Vector were also determined. Consistently, forced expression of NUDT21 significantly enhanced the mRNA and protein levels of SGPP2 in MKN-28 cells (**Figures 4E, G**). In addition, we detected the mRNA level of NUDT21 and SGPP2 in the 70 gastric cancer tissues, Pearson correlation analysis indicated a positive correlation between NUDT21 and SGPP2 expression in gastric cancer tissues (r=0.2890, *P*=0.0153) (**Figure 4H**). Therefore, NUDT21 positively regulated the expression of SGPP2 in human gastric cancer cells.

Besides, several other cell proliferation or apoptosis related genes (including c-Myc, Bcl-2, CCND1, CCNE1, p53 and PTEN) were also examined in NUDT21 shRNA transfected BGC-823 cells and NUDT21#OE plasmids transfected MKN-28 cells. As shown in **Supplementary Figure 1**, the mRNA levels of Bcl-2 and CCNE1 were much lower in BGC-823-sh-NUDT21#1/BGC-823-sh-NUDT21#2 cells compared with BGC-823-sh-ctrl cells and were much higher in MKN-28-NUDT21#OE cells compared with MKN-28-Vector cells. However, there were no significant expression level changes of c-Myc, CCND1, p53 or PTEN. Consistently, the protein levels (examined by western blot) of Bcl-2 and CCNE1 in BGC-823-sh-NUDT21#1/BGC-823-sh-NUDT21#2/BGC-823-sh-ctrl and MKN-28-NUDT21#OE/MKN-28-Vector cells showed the same trend as the mRNA levels (**Figures 4F, G**). Therefore, Bcl-2 and CCNE1 might contribute to the tumor promoting role of the NUDT21/SGPP2 pathway.

### NUDT21 Stimulated Cell Proliferation and Metastasis in Gastric Cancer Cells *via* Up-Regulating SGPP2

**Figure 5:**
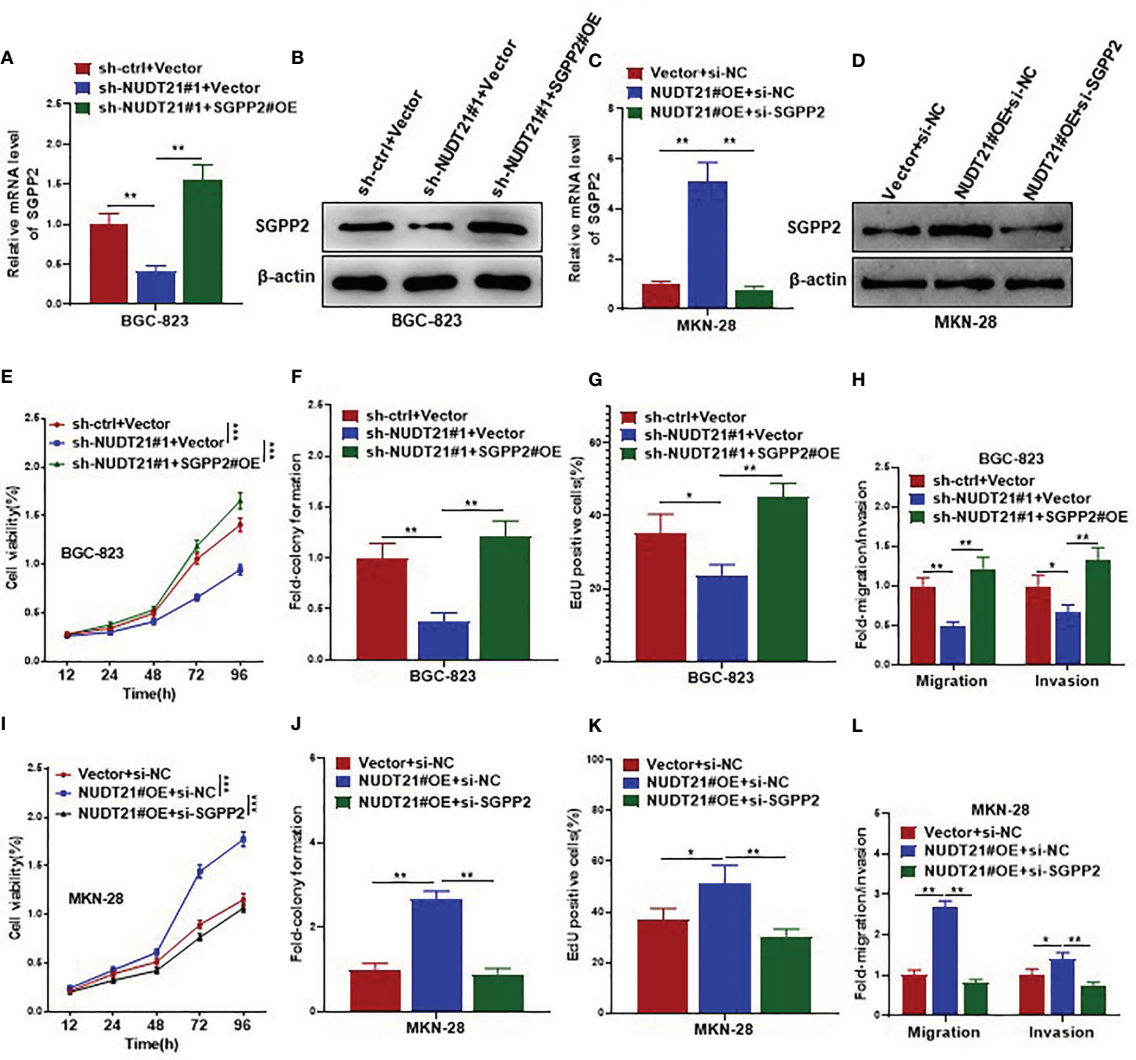
SGPP2 mediated the oncogenic role of NUDT21 in gastric cancer cells. BGC-823 cells were co-transfected with sh-NUDT21#1/sh-ctrl and SGPP2#OE/Vector, MKN-28 cells were co-transfected with NUDT21#OE/Vector and si-SGPP2/si-NC. **(A, C)** mRNA levels of SGPP2 were examined by RT-qPCR. GAPDH was used as control. **(B, D)** Protein levels of SGPP2 were examined by western blot. β-Actin was used as control. **(E, I)** MTT assay was carried out to detect cell viability. **(F, J)** Cell colony formation assay was performed to evaluate cell growth. **(G, K)** EdU assay was performed to evaluate cell proliferation. **(H, L)** Cell migration and invasion assays were carried out to detect cell metastasis. *P < 0.05; **P < 0.01; ***P < 0.001.

To examine whether the promoting role of NUDT21 in gastric cancer cells was mediated by SGPP2, rescue experiments were performed in BGC-823 and MKN-28 cells. As shown in **Figures 5A, B**, in BGC-823 cells, both mRNA and protein levels of SGPP2 decreased significantly after co-transfected with sh-NUDT21#1 and Vector, but these decreases were abolished by co-transfection with sh-NUDT21#1 and SGPP2 overexpressing plasmids (SGPP2#OE). Concordant with former results, cell viability (examined by MTT assay), cell colony formation (examined by cell colony formation assay), cell proliferation (examined by EdU assay), cell migration (examined by cell migration assay) and invasion (examined by cell invasion assay) all reduced significantly in BGC-823 cells after co-transfected with sh-NUDT21#1 and Vector. However, these decreases were abrogated by co-transfection with sh-NUDT21#1 and SGPP2#OE (**Figures 5E–H**).

On the other hand, in MKN-28 cells, both mRNA and protein levels of SGPP2 increased significantly after co-transfected with NUDT21#OE and negative control siRNA (si-NC), but these increases were abolished by co-transfection with NUDT21#OE and SGPP2 siRNA (si-SGPP2) (**Figures 5C, D**). Concordantly, MTT assay, cell colony formation assay, EdU assay, cell migration and invasion assays showed cell proliferation and metastasis increased significantly in MKN-28 cells after co-transfected with NUDT21#OE and si-NC. However, these increases were abrogated by co-transfection with NUDT21#OE and si-SGPP2 (**Figures 5I–L**).

In addition, we examined the role of SGPP2 in gastric cancer cells *in vivo*. As shown in **Supplementary Figure 2**, MKN-28-SGPP2#OE derived tumors showed faster growth, higher weight and more Ki-67 levels compared with MKN-28-Vector derived tumors *in vivo*; and more mice injected with MKN-28-SGPP2#OE cells (6 out of 8) were observed obvious lung micrometastasis compared with mice injected with MKN-28-Vector cells (2 out of 8) (*P*=0.0455).

Therefore, NUDT21 stimulated cell proliferation and metastasis in gastric cancer cells through specific regulation of SGPP2.

## Discussion

In this study, we systematically examined the functional role of NUDT21 in human gastric cancer cells. The expression levels of NUDT21 were much higher in human gastric cancer tissues/cells compared with normal gastric tissues/cells. Gastric cancer tissues from patients with distant metastasis showed an elevated NUDT21 level compared with the tissues from patients without distant metastasis. High expression level of NUDT21 was associated with tumor size, lymph node metastasis and clinical stage in gastric cancer patients, and patients with high expression of NUDT21 showed poor OS rates compared with patients with low expression of NUDT21. shRNA mediated depletion of NUDT21 dramatically decreased cell viability, cell colony formation, cell proliferation, cell migration and invasion in gastric cancer cells as determined by MTT assay, cell colony formation assay, EdU assay, cell migration assay and cell invasion assay respectively. Forced expression of NUDT21 concordantly promoted both cell proliferation and metastasis in gastric cancer cells. Moreover, in animal experiments, forced expression of NUDT21 promoted tumor growth and cell proliferation in gastric cancer cells, and depletion of NUDT21 restrained lung metastasis of gastric cancer cells. As reported previously, NUDT21 played an oncogenic role in human pancreatic ductal adenocarcinoma cells, promoting both cell proliferation and metastasis (12). Pancreatic ductal adenocarcinoma tissues showed elevated NUDT21 compared with normal tissues, and high level of NUDT21 predicted poor prognosis of patients with pancreatic ductal adenocarcinoma (12). In leukemic cells, depletion of NUDT21 was reported to inhibit cell proliferation and promote cell apoptosis (13, 14). The NUDT21 mRNA levels were much higher in patients with primary chronic myelocytic leukemia compared with normal control (13, 14). These published results were consistent with our current findings. However, NUDT21 was identified to be a tumor suppressor in human cervical cancer (22), bladder cancer (23), lung cancer (including small cell lung cancer and non-small cell lung cancer) (24–26), breast cancer (16, 27, 28), and hepatocellular carcinoma (15, 29, 30). Moreover, the role of NUDT21 in human glioma was controversial: Jia-Cheng et al. reported that NUDT21 was up-regulated in human glioma tissues, and NUDT21 promoted the proliferation of glioma cells through the NF-κB signaling pathway (31); in Chu Y et al.’s study, they identified that NUDT21 regulated the alternative polyadenylation of Pak1 and reduced expression of NUDT21 predicted worse survival in low grade glioma and glioblastoma patients (32). These results demonstrated that NUDT21 had tissue specificity in different kinds of human cancers.

For downstream mechanisms, SGPP2 was identified to be positively regulated by NUDT21 in gastric cancer cells. Forced expression of SGPP2 rescued the decrease of cell proliferation and metastasis induced by NUDT21 depletion in BGC-823 cells; depletion of SGPP2 rescued the increase of cell proliferation and metastasis induced by NUDT21 in MKN-28 cells. Therefore, SGPP2 mediated the tumor promoting role of NUDT21 in gastric cancer cells. As reported previously, increased expression of SGPP2 promoted cell proliferation, survival, invasion and tumor angiogenesis in glioblastoma by promoting the soluble sphingolipid metabolite sphingosine 1-phosphate (S1P) (33). The 5-methylcytosine correlation score composed of SGPP2 and 6 other genes was reported to be significantly related to the prognosis of hepatocellular carcinoma (34). These results were concordant with our present results. Besides, SGPP2 was involved in sphingolipid metabolism, and the defect of sphingolipid metabolism was related to the pathogenesis of SLE (35); induction of SGPP2 expression contributed to the pathogenesis of colitis by promoting the destruction of mucosal barrier function (36); β cell endoplasmic reticulum stress caused by the loss of SGPP2 facilitated the development of diabetes (37). We herein reported that SGPP2 was specifically regulated by NUDT21, promoting gastric cancer cell proliferation and metastasis, and mediated the oncogenic role of NUDT21 in gastric cancer cells. In addition, we have examined that Bcl-2 and CCNE1 were positively regulated by NUDT21. Bcl-2 and CCNE1 were famous factors contributing to cell proliferation in nearly all kinds of human cancers (38–41). Moreover, Lan Zhang et al. have reported that both Bcl-2 and CCNE1 were positively regulated by NUDT21 and they mediated the promoting role of NUDT21 in human leukemia cells (13). These results were consistent with our present study and supported our results. Therefore, Bcl-2 and CCNE1 might contribute to the tumor promoting role of the NUDT21/SGPP2 pathway in gastric cancer cells. For detailed mechanism involved, it should be studied in further.

In summary, we have demonstrated the oncogenic role of NUDT21 in human gastric cancer cells. Gastric cancer tissues (especially with tumor metastasis) expressed high level of NUDT21. High level of NUDT21 was correlated with poor clinicopathological features and survival rates in gastric cancer patients. SGPP2 was positively regulated by NUDT21 and mediated the oncogenic role of NUDT21 in gastric cancer cells. NUDT21 could be used as a potential diagnostic and therapeutic target for human gastric cancer.

## Data Availability Statement

The datasets presented in this study can be found in online repositories. The names of the repository/repositories and accession number(s) can be found in the article/**Supplementary Material**.

## Ethics Statement

The animal study was reviewed and approved by the Ethics Committee of Animal Experiments of Anhui Medical College.

## Author Contributions

YoZ participated in study design and performed cell functional experiments. RZ performed cell functional experiments and xenograft analysis. YiZ performed western blot and RT-qPCR. XC participated in cell functional experiments. LL performed immunohistochemistry. ZW participated in data analysis and manuscript revision. KD participated in study design and writing. All authors contributed to the article and approved the submitted version.

## Funding

This work was supported in part by grants from the Natural Science Foundation of Anhui Province (2008085QH378, 2008085MH276, 2008085QH411), National Nature Science Foundation of China (81972472), and Basic and Clinical Cooperative Research Promotion Program of Anhui Medical University (2020xkjT013).

## Conflict of Interest

The authors declare that the research was conducted in the absence of any commercial or financial relationships that could be construed as a potential conflict of interest.

## Publisher’s Note

All claims expressed in this article are solely those of the authors and do not necessarily represent those of their affiliated organizations, or those of the publisher, the editors and the reviewers. Any product that may be evaluated in this article, or claim that may be made by its manufacturer, is not guaranteed or endorsed by the publisher.
