# Corrigendum: NUDT21 promotes tumor growth and metastasis through modulating SGPP2 in human gastric cancer

**DOI:** 10.3389/fonc.2023.1240063

**Published:** 2023-08-28

**Authors:** Yong Zhu, Rumeng Zhang, Ying Zhang, Xiao Cheng, Lin Li, Zhengsheng Wu, Keshuo Ding

**Affiliations:** ^1^Department of Pathophysiology, School of Basic Medicine, Anhui Medical University, Hefei, China; ^2^Department of Pathology, School of Basic Medicine, Anhui Medical University, Hefei, China; ^3^Department of Oncology of the First Affiliated Hospital, Division of Life Science and Medicine, The CAS Key Laboratory of Innate Immunity and Chronic Disease, University of Science and Technology of China, Hefei, China; ^4^Department of Pathology, The First Affiliated Hospital of Anhui Medical University, Hefei, China

**Keywords:** NUDT21, SGPP2, gastric cancer, proliferation, metastasis

**Error in Figure/Table Legend**

In the published article, there was an error in the legend for (**Figure 1F**) as published.

This sentence previously stated: “(F) mRNA levels of NUDT21 in 31 gastric cancer tissues from patents with metastasis and 39 gastric cancer tissues from patients without metastasis were analyzed by RT-qPCR”.

The corrected sentence appears below:

(F) mRNA levels of NUDT21 in 31 gastric cancer tissues from patients with distant metastasis and 39 gastric cancer tissues from patients without distant metastasis were analyzed by RT-qPCR.

The authors apologize for this error and state that this does not change the scientific conclusions of the article in any way. The original article has been updated.

**Figure 1:**
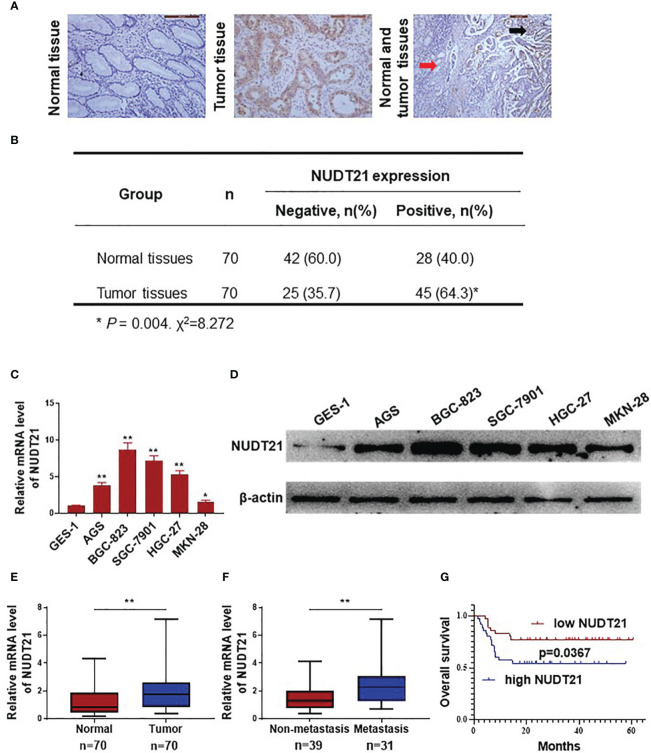
Expression of NUDT21 in human gastric cancer/normal gastric tissue samples and cell lines. **(A, B)** Protein levels of NUDT21 in 70 gastric cancer tissues and 70 normal gastric tissues were examined by immunohistochemistry. The magnifications were 200 and 100 respectively. Red arrows: normal gastric tissue; black arrows: gastric cancer tissue. **(C)** Relative mRNA levels of NUDT21 in gastric cancer cells AGS, BGC-823, SGC-7901, HGC-27, MKN-28 and normal gastric cell GES-1 were examined by RT-qPCR. GAPDH was used as control. **(D)** Protein levels of NUDT21 in gastric cancer cells AGS, BGC-823, SGC-7901, HGC-27, MKN-28 and normal gastric cell GES-1 were examined by western blot. β-Actin was used as control. **(E)** mRNA levels of NUDT21 in 70 gastric cancer tissues and 70 normal gastric tissues were examined by RT-qPCR. **(F)** mRNA levels of NUDT21 in 31 gastric cancer tissues from patients with distant metastasis and 39 gastric cancer tissues from patients without distant metastasis were analyzed by RT-qPCR. **(G)** Kaplan-Meier curves showed the overall survival (OS) rates in gastric cancer patients with high and low levels of NUDT21. *P < 0.05; **P < 0.01.

**Text Correction**

In the published article, there was an error.

A correction has been made to **Abstract**, paragraph one. This sentence previously stated:

“The expression levels of NUDT21 were also much higher in gastric cancer tissues from patients with tumor metastasis compared with those of patients without tumor metastasis.”

The corrected sentence appears below:

“The expression levels of NUDT21 were also much higher in gastric cancer tissues from patients with distant metastasis compared with those of patients without distant metastasis.”

The authors apologize for this error and state that this does not change the scientific conclusions of the article in any way. The original article has been updated.

**Text Correction**

In the published article, there was an error.

A correction has been made to **Introduction**, paragraph three. This sentence previously stated:

“The expression levels of NUDT21 were also higher in gastric cancer tissues from patients with tumor metastasis compared with the tissues from patients without tumor metastasis.”

The corrected sentence appears below:

“The expression levels of NUDT21 were also higher in gastric cancer tissues from patients with distant metastasis compared with the tissues from patients without distant metastasis.”

The authors apologize for this error and state that this does not change the scientific conclusions of the article in any way. The original article has been updated.

**Text Correction**

In the published article, there was an error.

A correction has been made to **Results**, *Expression of NUDT21 in Human Gastric Cancer/Normal Gastric Tissue Samples and Cell Lines*, paragraph one. This sentence previously stated:

“Moreover, the mRNA levels of NUDT21 were dramatically higher in gastric cancer tissues from patients with tumor metastasis compared with gastric cancer tissues from patients without tumor metastasis (**Figure 1F**).”

The corrected sentence appears below:

“Moreover, the mRNA levels of NUDT21 were dramatically higher in gastric cancer tissues from patients with distant metastasis compared with gastric cancer tissues from patients without distant metastasis (**Figure 1F**).”

The authors apologize for this error and state that this does not change the scientific conclusions of the article in any way. The original article has been updated.

**Text Correction**

In the published article, there was an error.

A correction has been made to **Discussion**, paragraph one. This sentence previously stated:

“Gastric cancer tissues from patients with tumor metastasis showed an elevated NUDT21 level compared with the tissues from patients without tumor metastasis.”

The corrected sentence appears below:

“Gastric cancer tissues from patients with distant metastasis showed an elevated NUDT21 level compared with the tissues from patients without distant metastasis.”

The authors apologize for this error and state that this does not change the scientific conclusions of the article in any way. The original article has been updated.

