# Supplementary figures and images for: NUDT21 Promotes Tumor Growth and Metastasis Through Modulating SGPP2 in Human Gastric Cancer
Source: Front Oncol. 2021 Sep 30;11:670353. doi: 10.3389/fonc.2021.670353 (PMC8514838; doi:10.3389/fonc.2021.670353)

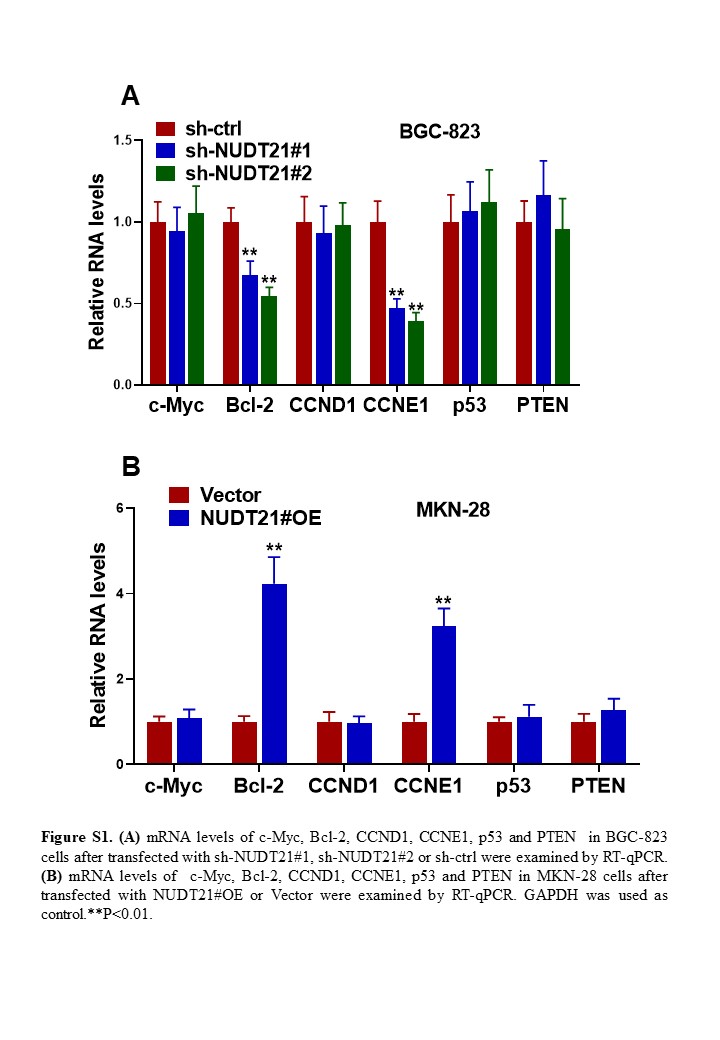

Supplement: Supplementary file 1 [file Image_1.jpeg]

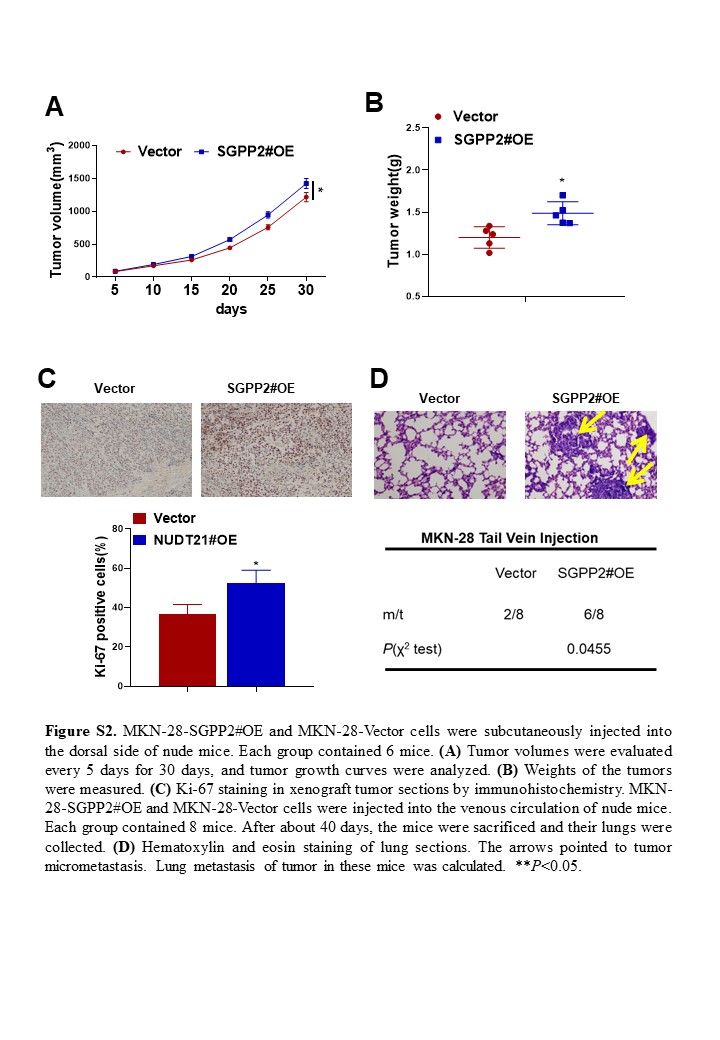

Supplement: Supplementary file 2 [file Image_2.jpeg]

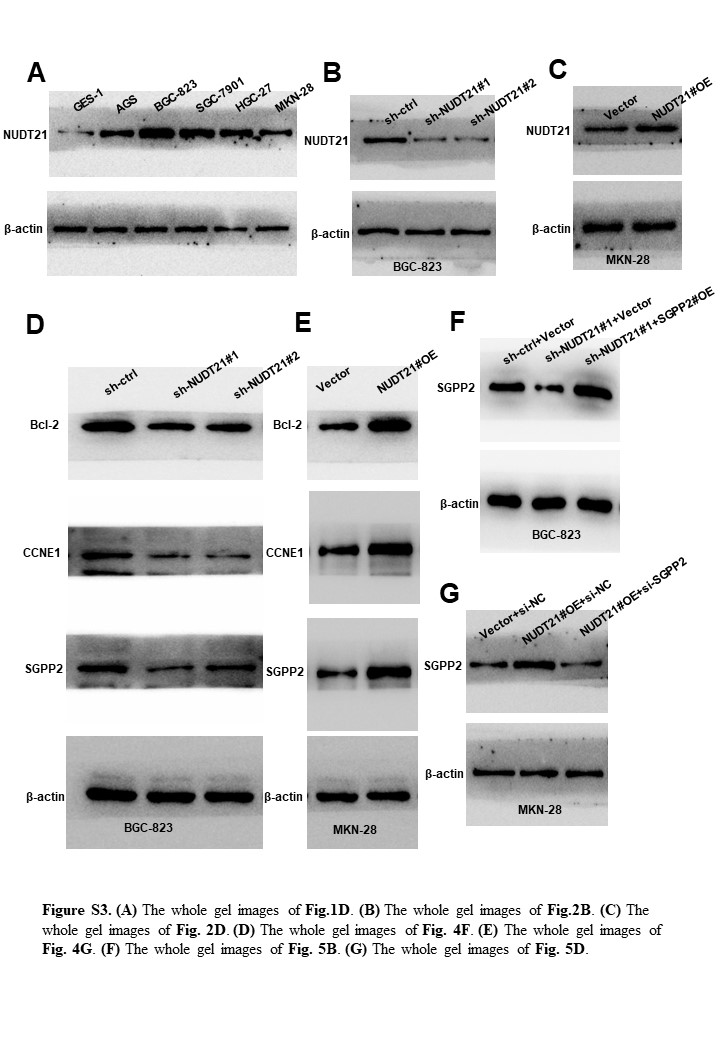

Supplement: Supplementary file 3 [file Image_3.jpeg]
